# Flavin-Dependent Enzymes in Cancer Prevention

**DOI:** 10.3390/ijms131216751

**Published:** 2012-12-07

**Authors:** Danuta Wojcieszyńska, Katarzyna Hupert-Kocurek, Urszula Guzik

**Affiliations:** Department of Biochemistry, Faculty of Biology and Environmental Protection, University of Silesia in Katowice, Jagiellonska 28, 40-032 Katowice, Poland; E-Mails: danuta.wojcieszynska@us.edu.pl (D.W.); khupert@us.edu.pl (K.H.-K.)

**Keywords:** flavin-containing monooxygenase, methylenetetrahydrofolate reductase, monoamine oxidase

## Abstract

Statistical studies have demonstrated that various agents may reduce the risk of cancer’s development. One of them is activity of flavin-dependent enzymes such as flavin-containing monooxygenase (FMO)_GS-OX1_, FAD-dependent 5,10-methylenetetrahydrofolate reductase and flavin-dependent monoamine oxidase. In the last decade, many papers concerning their structure, reaction mechanism and role in the cancer prevention were published. In our work, we provide a more in-depth analysis of flavin-dependent enzymes and their contribution to the cancer prevention. We present the actual knowledge about the glucosinolate synthesized by flavin-containing monooxygenase (FMO)_GS-OX1_ and its role in cancer prevention, discuss the influence of mutations in FAD-dependent 5,10-methylenetetrahydrofolate reductase on the cancer risk, and describe FAD as an important cofactor for the demethylation of histons. We also present our views on the role of riboflavin supplements in the prevention against cancer.

## 1. Introduction

Riboflavin, 7,8-dimethyl-10-ribityl-isoalloxazine, is a water-soluble vitamin present in a wide variety of foods, such as milk, offal, cereals or dark-green leafy vegetables [1,2]. It is well established that riboflavin participates in a diversity of redox reactions which absolutely play a key role in the function of human cells, through the cofactors FMN and FAD, which act as electron carriers. Insufficient intake of riboflavin can lead to disturbances in reactions of intermediary metabolism, together with the functional implications [1]. It was also demonstrated that riboflavin can play an important role in the development and progression of cancer [3]. On the other hand, it can protect against cancer or support chemotherapy [4–6].

In connection with an increasing interest in the role of riboflavin in protecting against cancer it was important and necessary to present the actual knowledge about the relationship between riboflavin intake, flavin-dependent enzyme, and the risk of cancer.

## 2. Role of Flavin-Containing Monooxygenase in Glucosinolate Synthesis

Glucosinolates (GSLs) are sulfur-rich secondary plant metabolites containing β-thioglucopyranose residue and *N*-hydroxyiminosulfate ester which form the core linked to a structurally diverse amino acid-derived side chain [7]. Based on their amino acid precursors GSLs are classified into three major groups: aliphatic glucosinolates, derived from Ala, Leu, Ile, Val, and Met; benzenic glucosinolates, derived from Phe or Tyr, and indolic glucosinolates, derived from Trp [8,9]. The most abundant of all GSLs is indole glucosinolate which is found in high concentration in cruciferous vegetables such as broccoli, cabbage, cauliflower or Brussels sprouts [7,10–12]. Epidemiological studies have shown that a diet rich in glucosinolates correlates with a decreased risk of developing many common cancers [10–14]. The anticarcinogenic effect of GSLs is probably associated with the properties of indoles and isothiocyanates, the certain products of glucosinolates hydrolysis. These compounds are known to influence the activity of phase I and II biotransformation enzymes involved in the modulation of metabolism of carcinogenic and/or mutagenic compounds, thereby preventing the formation of electrophilic intermediates which can bind to DNA [15–17]. The key enzyme in glucosinolate biosynthesis is flavin-dependent monooxygenase and activity of this enzyme as well as flavin levels in plant indirectly influences the risk of cancer in humans.

Biosynthesis of glucosinolate generally occurs through three independent stages: chain elongation of selected precursor amino acid; formation of the core structure and secondary modification of the aliphatic chain [7–9]. Initial reaction in GSLs formation, transamination takes place in the cytosol. Side chain elongation occurs in the chloroplast and the core structure formation is catalyzed by enzymes associated with the endoplasmatic reticulum membranes as well as those sited in the cytosol [8]. However not much information is known about the localization of the final step in GSL biosynthesis, and the side chain modifications of the glucosinolate are of particular importance because the biological activity of the glucosinolate hydrolysis products is determined to a large extent by the structure of the side chain [14]. Additionally, secondary modifications together with the side-chain elongation are responsible for more than 120 known glucosinolate structures [8].

**Figure 1:**
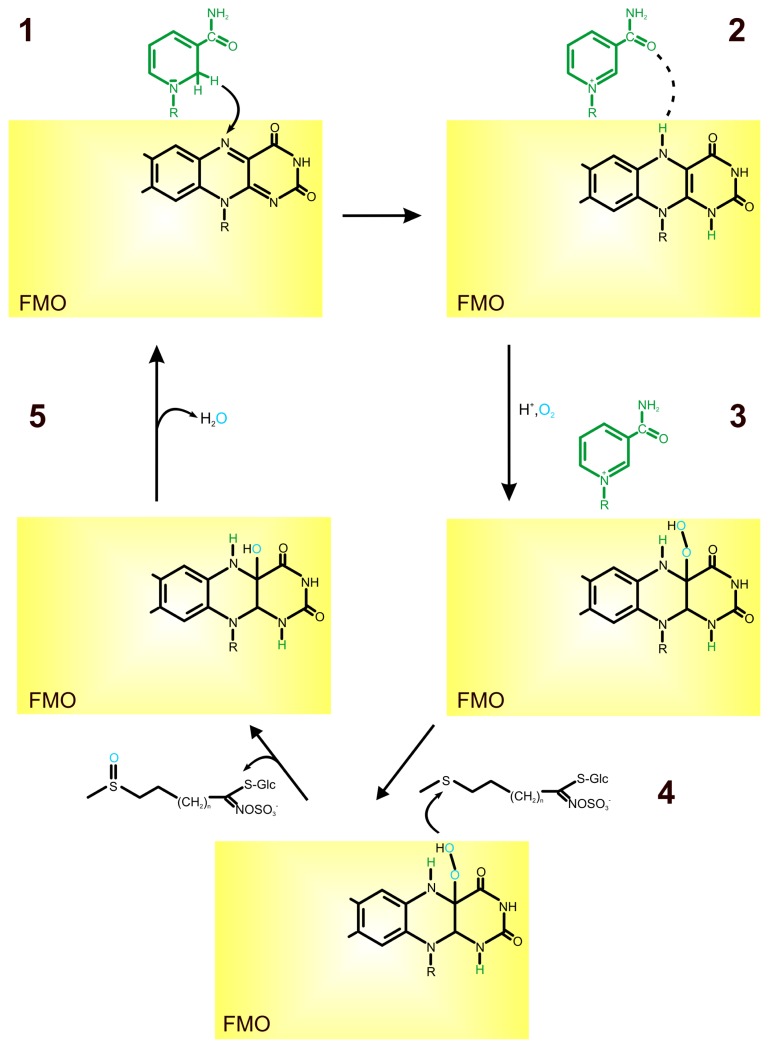
Role of FAD in *S*-oxygenation of glucosinolate. FAD is reduced to FADH_2_ through a hydride ion transfer from NADPH (**1**, **2**); FADH_2_ binds molecular oxygen at the 4α position of the isoalloxazine ring resulting in a 4α-hydroperoxyflavin adenine dinucleotide (FAD-OOH) formation (**3**); when a suitable substrate, such as S in methylthioalkyl, binds to the protein/FAD-OOH complex it is oxygenated to SO through the OOH moiety (**4**); the oxygen atom reacts to form water, NADP is released and binding of another NADPH starts a new cycle (**5**) [11,18,19].

Plant flavin-containing monooxygenases (FMOs) are proteins of about 50 kDa with the FMO-identifying motif (“FxGxxxHxxxY/F”) and the conserved “GxGxxG” motif for binding FAD and NADPH. FMOs catalyze the transfer of one atom of molecular O_2_ to a large variety of small, nucleophilic compounds, among them sulfur-containing substrates, thus rendering them more polar [18–20]. The catalytic activity of these enzymes is carried out mainly through the prosthetic group FAD and cofactor NADPH. FAD is the major electron carrier in the oxidation-reduction processes catalyzed by various enzymes. The electron donor in the *S*-oxygenation of methylthioalkyl is NADPH [19]. In the catalytic cycle, FAD is first reduced to FADH_2_ through a hydride ion transfer from NADPH. In the second step FADH_2_ binds molecular oxygen at the 4α position of the isoalloxazine ring, resulting in a 4α-hydroperoxyflavin adenine dinucleotide (FAD-OOH) formation [19,20]. The hydride ion transfer from NADPH to isoalloxazine ring was proposed by Eswaramoorthy *et al.*[19]. According to their assumptions the nicotinamide portion of NADPH stacks with the flavin of FAD in a novel fashion. Atom C2 of the nicotinamide base is 3.35 Å from N5 of the flavin ring and the hydride ion transfer takes place through these two atoms. The N5 atom of the reduced flavin moiety makes a hydrogen bond with O7 of the nicotinamide. In the enzyme-FAD-NADPH complex the prostetic group exists as a FADH_2_ and the cofactor is in the protonated form as a NADP^+^. In this state, the FADH_2_ is ready to bind molecular oxygen. This molecular oxygen is located near the flavin ring and is readily available for 4α-hydroperoxyflavin formation [19]. The isoalloxazine moiety attached to the proximal peroxy-oxygen is electron-withdrawing, polarizing the O–O bond and activating it for heterolytic cleavage [20]. In this conformation, the enzyme awaits the *S*-containing substrate, which needs a free electron pair which can enter into the active site. When a suitable substrate with a nucleophilic atom, such as the S in methylthioalkyl, binds productively to the protein/FAD-OOH complex, it is oxygenated to SO through the OOH moiety. The other oxygen atom reacts to form water. NADP is released and the binding of NADPH starts a new cycle (Figure 1) [18,19].

Flavin-containing monooxygenases (FMO)_GS-OX_, have been identified in *Arabidopsis* as enzymes catalyzing the first step in side chain modification of aliphatic glucosinolates, the *S*-oxygenation from methylthioalkyl to methylsulphinylalkyl GSLs. According to phylogenetic analysis, FMO_GS-OX_ are assigned to Clade III of plant FMOs phylogenetic tree [11,12,14]. Within enzymes of Clade III the characteristic amino acid motif “HCTGYK” was also found [18]. Li *et al.*[14] analyzed subcellular localization of these enzymes by fusing the FMO_GS-OX_ with the yellow fluorescent protein (YFP). However the catalytic function of this enzyme was observed in cytosol, YFP signals were also detected in the ER. Authors suggested that the ER-located fluorescence signal was connected with the early phase of biosynthesis or transportation of FMO_GS-OX_[14].

Plant FMOs do not transform physiological nucleophiles, *i.e.*, methionine, cysteine and glutathione and do not show such a broad substrate specificity as the animal FMOs [11,18]. Hansen *et al.*[11] have shown that the *S*-oxygenating activity of FMO_GS-OX_ from *Arabidopsis* is selective for potential substrates containing a methylthiol moiety. Moreover, the enzyme product is a pure epimer and its sulfoxide group has the RS configuration which indicates that the enzyme is stereospecific [7].

## 3. Relationship between FAD-Dependent 5,10-Methylenetetrahydrofolate Reductase and Cancer Risk

5,10-Methylenetetrahydrofolate reductase (MTHFR ) is present in both prokaryotic and eukaryotic organisms [21–27] and the sequences of human methylenetetrahydrofolate reductase show clear homology with their bacterial analogs [28]. MTHFR catalyze the NAD(P)H-dependent reduction of 5,10-methylenetetrahydrofolate to 5-methyltetrahydrofolate.

In this reaction, MTHFR transfers a hydride ion from the nicotinamide C4 to methylenetetrahydrofolate using a flavin adenine dinucleotide N5 atom as an intermediate hydride acceptor and donor [26]. MTHFR is the only enzyme known to catalyze the direct exchange of hydride between folate and FAD [22,25,26,29]. It was shown that the bacterial enzyme-bound flavin was reduced by NADH, and much more slowly by NADPH while the mammalian enzymes use NADPH [24,26,28]. Reduction of 5,10-methylenetetrahydrofolate to 5-methyltetrahydrofolate creates tetrahydrofolate-bound one-carbon units used in the methylation of homocysteine to form methionine, the terminal step in methionine biosynthesis [21–26,28–30].

5,10-Methylenetetrahydrofolate reductase is a multicomponent enzyme consisting of 2–4 subunits, each of them containing a molecule of non-covalently bound flavin adenine dinucleotide [21,24,26,28,29,31]. Human MTHFR gene has been localized at the end of the short arm of chromosome 1 (1p36.3). It encodes a polypeptide of 77 kD which forms homodimers of about 150 kDa [22,29,32]. Each subunit of MTHFR comprises a catalytic and a regulatory domain. The 40 kDa catalytic region of the human MTHFR is located within the *N*-terminal domain. It contains determinants for the binding of FAD, NAD(P)H and methylenetetrahydrofolate [28,32]. The regulatory region (37 kDa) of MTHFR, which is responsible for binding of adenosylmethionine/adenosylhomocysteine (AdoMet/AdoHcy) is located at the *C*-terminal fragment of the molecule. It was shown that MTHFR activity is allosterically regulated by the AdoMet/AdoHcy ratio, with AdoMet as an inhibitor and AdoHcy competing with AdoMet for binding to the enzyme but not acting as an inhibitor [28,32]. If the cell is repleted with AdoMet and the ratio of AdoMet/AdoHcy is high, then the MTHFR is inhibited [29,33]. Both catalytic and regulatory domains of human MTHFR are connected by an extremely hydrophilic region (Lys-Arg-Arg-Glu-Glu-Asp) [28,32].

There are two well-described, frequently occurring polymorphisms in the MTHFR gene: c.*677C* > *T* and c.*1298A* > *C* (GenBank accession number U09806). The c.*677C* > *T* polymorphism lies at the base of the binding site for the FAD. It causes an alanine-to-valine substitution, producing a thermolabile form of the enzyme, and has been associated with reduced enzyme activity [29,34,35]. The thermolabile form of the MTHFR enzyme has been shown to dissociate with the FAD cofactor more readily (approximately 10 times) than wild-type enzyme [3,29,31,36,37]. c.*1298A* > *C* polymorphism is located in exon 7 coding the *S*-adenosylmethionine-regulatory domain of the enzyme. It results in a glutamate-to-alanine substitution at codon 429 (E429A).

**Figure 2:**
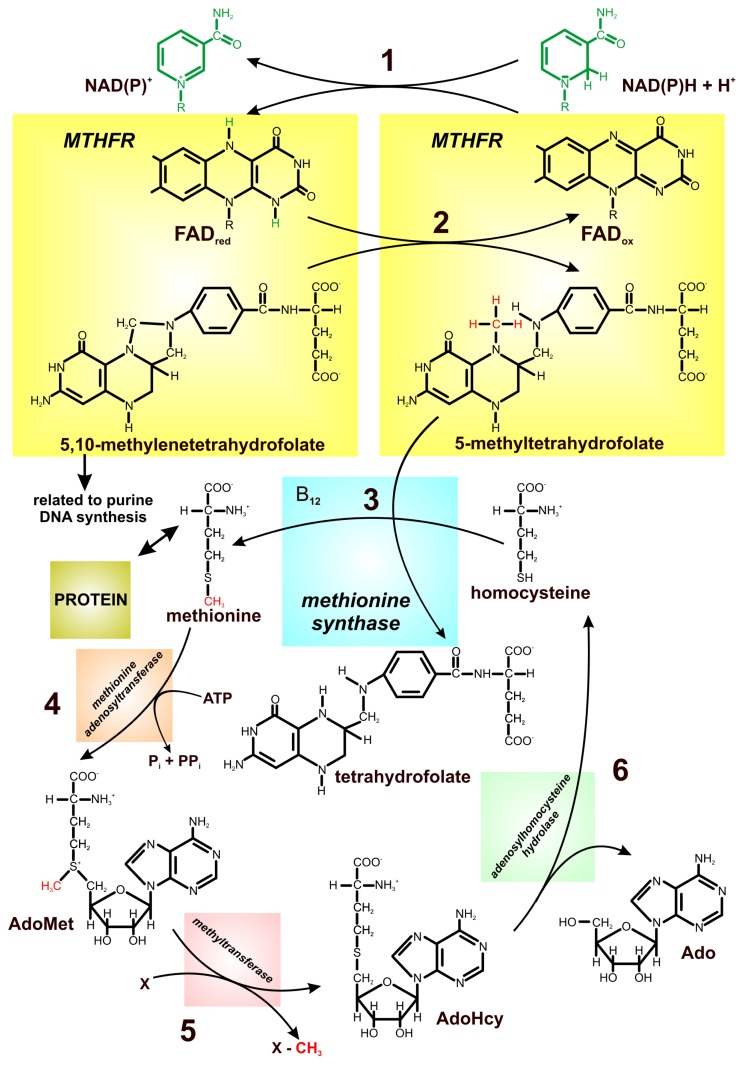
Schematic illustration of the one-carbon metabolism/pathways noting the cross-talk between DNA synthesis and methylation processes. MTHFR (5,10-methylenetetrahydrofolate reductase) transfers a hydride ion from the nicotinamide to methylenetetrahydrofolate using flavin adenine dinucleotide as an intermediate hydride acceptor and donor (**1**, **2**); reduction of 5,10-methylenetetrahydrofolate to 5-methyltetrahydrofolate commits tetrahydrofolate-bound one-carbon units to use in the methylation of homocysteine to form methionine (**3**); methionine is converted to adenosylmethionine (AdoMet), which serves as a methyl donor for biosynthetic reaction (**4**, **5**); adenosylhomocysteine (AdoHcy) is hydrolyzed to adenosine and homocysteine (**6**) [26,27,40], modified.

MTHFR is one of the key enzymes in one-carbon metabolism in which homocysteine, a nonprotein forming, sulfur-containing amino acid plays a crucial role (Figure 2). MTHFR regulates the flow of folates between the production of nucleotides and the supply of methyl groups for methionine synthesis [38,39]. As it was previously mentioned, riboflavin is the precursor of FAD which serves as the cofactor for MTHFR and, therefore, riboflavin deficiency may reduce the enzyme activity. Reduced ability of the enzyme to catalyze the conversion of 5,10-methylenetetrahydrofolate to 5-methyltetrahydrofolate leads to hyperhomocysteinemia [27,29]. Elevated plasma homocysteine concentrations are associated with several metabolic disorders, including high body mass index, high plasma triglyceride levels, hypertension, and abnormal oxidation of low density lipoprotein, which may lead to the development of a wide variety of cancers, such as breast, ovarian, and pancreatic cancers [40,41]. Additionally, the activity of MTHFR is also a determinant of the availability of folate for DNA methylation.

Any disturbance in homocysteine metabolism disturbs cellular methylation processes and DNA hypomethylation is a feature of early tumorgenesis and is associated with cancer development. Plasma total homocysteine (tHcy) concentration is a responsive indicator of B2, B6, B12 and MTHFR status [38]. In patients with hyperhomocysteinemia, increased concentration of homocysteine thiolactone, which can react with lysine residues and free amine groups on numerous cellular proteins, was observed [40,42].

Numerous studies have shown that polymorphisms in the gene-encoding methylenetetrahydrofolate reductase may impede homocysteine remethylation [34,39]. A common mutation (*c.677C* > *T)* described earlier, has been identified with a frequency of 0.32 in the Caucasian population. Individuals homozygous for this mutation (*TT*) have about 30%–35% of the normal MTHFR activity.

**Figure 3:**
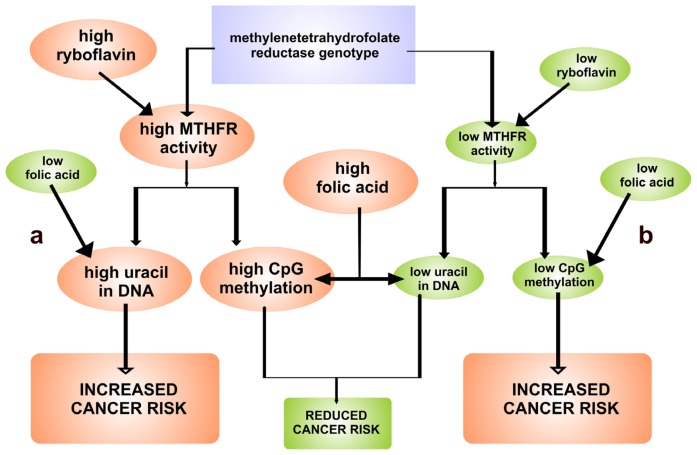
The interrelationship between methylenetetrahydrofolate reductase (MTHFR) genotype, riboflavin and folic acid with respect to (**a**) cytosine-phosphate-guanosine dinucleotide (CpG) methylation and uracil in DNA; (**b**) initiation of cancer caused by CpG hypomethylation. The level of riboflavin and folic acid depends on supplementation [3], modified.

The homocysteine remethylation catalyzed by methionine synthase involves its conversion to methionine by the transfer of a methyl group from 5-methyltetrahydrofolate, the major circulatory form of folate, and is thought to be the most important reaction affecting plasma tHcy [39–41,43]. The relationship between riboflavin status and tHcy concentration is confined mainly to subjects with the *T* allele but not in subjects with the *CC* genotype. It has been shown by various authors [3,29,31,36,37] that the thermolabile form of the MTHFR enzyme dissociates with FAD cofactor more readily than wild-type enzyme. Therefore, subjects with *T* allele may require higher concentration of FAD for maximal catalytic activity. This may explain an increased level of tHcy at low levels of riboflavin. It was suggested that riboflavin deficiency causes an increased oxidation state of the folate pool and a reduction in the relative amounts of 5-methyltetrahydrofolate [44]. Both riboflavin and folate can protect against loss of function of MTHFR and, in consequence, can reduce the risk of cancer (Figure 3) [45].

Riboflavin intake was a determinant of total homocysteine in men and women in the Framingham Offspring Cohort. In individuals with the *TT* genotype and a low folate status from the same population, total homocysteine was inversely proportional to plasma riboflavin concentration [31,45]. On the base of these results it has been postulated that riboflavin status may influence on the metabolism of reduced folates, particulary in individuals with the *MTHFR T* variant. Individuals with *TT* and, possibly, *CT* genotypes may require a higher level in cytoplasm both folate and riboflavin to overcome the partial metabolic block and retain low total homocysteine concentrations [3,44,45].

Studies on riboflavin-deficient populations are needed to evaluate the utility of riboflavin supplementation in hyperhomocysteinemia [46].

## 4. FAD as an Important Cofactor for the Demethylation of Histones

Histones pack the eukaryotic DNA into the nucleosome, basic structural unit of chromatin. These alkaline proteins, and, in particular, their *N*-terminal tails, are substrates for a number of covalent post-translational modifications which are involved in chromatin remodeling and closely linked to transcriptional regulation, DNA replication, and DNA repair [47–50]. Histone acetylation and methylation of the lysine and arginine residues represent the most common modifications [48,51]. The methylation of lysine is one of the most extensively characterized epigenetic marks involved in the regulation of fundamental processes, such as heterochromatin formation, X-chromosome inactivation, and DNA repair [50,52]. Each of the methylated lysine residues can be un-, mono-, di-, or trimethylated, and the extent of methylation at a specific residue is important for the recognition of effector proteins and thus has impact on chromatin and the transcriptional outcome [48,51].

Histone lysine modification is a dynamic process established by specific lysine methyltransferases and demethylases interactions. Until now, two families of enzymes involved in demethylation of methylated lysine residues have been described in humans. The first class comprises jumonji domain-containing (JmjC) demethylases, while the second is represented by the amine oxidase domain-containing enzymes LSD1 and LSD2, also known as KDM1A and KDM2B. LSD1/2 catalyse demetylathion of mono- and dimethylated lysine 4 of histone H3 (H3K4) [49,53–56] but not trimethyllysine residues, as the quaternary ammonium group cannot form the requisite imine intermediate [57]. Despite the fact the same H3-Lys4 demethylase activity, LSD2 functions in distinct transcriptional compexes with specific biological functions [58].

LSD1 and LSD2 are flavin-dependent amine oxidases closely related to the polyamine oxidase superfamily [52,53,56]. LSD1 is an asymmetric molecule consisting of an *N*-terminal SWIRM domain (named for its presence in the proteins Swi3, Rsc8, and Moira) and *C*-terminal an AOL (amine oxidase-like) domain containing sites to bind the methylated substrate and FAD cofactor [47,50,52,53,59]. *N*- and *C*-terminal domains of LSD1 form a globular core structure from which the third domain, named Tower domain, protrudes as an elongated helix-turn-helix motif [59]. This domain is important for the histone demethylase activity of LSD1 and may also act as an adaptor to recruit other proteins [60]. According to Chen *et al.*[60] SWIRM domain of LSD1 is composed of five α-helices surrounding a central long helix, SWα4, and additional two-stranded β-sheet formed between the SWα4- SWα5 loop and the C terminus of the SWIRM domain [60]. This *N*-terminal motif important for protein stability, is thought to be responsible for the association of the enzyme with a variety of transcriptional protein complexes, protein–protein and DNA–protein interactions [49]. *C*-terminal amine oxidase-like domain of this protein folds into FAD-binding subdomain and a substrate-binding subdomain, thereby creating a big cavity which defines the enzyme activity center at their interface. The FAD-binding subdomain shows a mixed α-β structure while the substrate-binding subdomain is characterized by a six-stranded mixed β-sheet flanked by six α-helices [49,60]. The large cavity of the catalytic center of LSD1 is framed by many structural elements which surround the catalytic center. A six-stranded β-sheet and a long helix form its left side and the bottom surfaces, respectively, while a short one-turn helix together with a two-stranded β-sheet, and several loops cover the rest of the cavity. The left side of the catalytic cavity is flat and composed mainly of hydrophobic residues. Its right side presents mainly the acidic side chains and the backbone carbonyl oxygen atoms on the concave-shaped surface [60]. The crystal structure of LSD1 revealed a series of hydrogen bond and van der Waals interactions between the side chains of H3 residues and those of the LSD1 active site [52,58].

Although LSD1 and LSD2 share a similar catalytic domain, they differ in the domains which are involved in protein–protein interactions [50,55]. In contrast to LSD1, LSD2 lacks the Tower domain which is instrumental to the formation of the complex of LSD1 with its corepressor protein, CoREST. Moreover, it contains a CW-type zinc finger motif with potential zinc-binding sites in its *N*-terminal region, which is not present in LSD1. It is suggested that this unique structure of LSD2 may confer its biochemical and biological properties as distinct from those of LSD1 [50,61].

**Figure 4:**
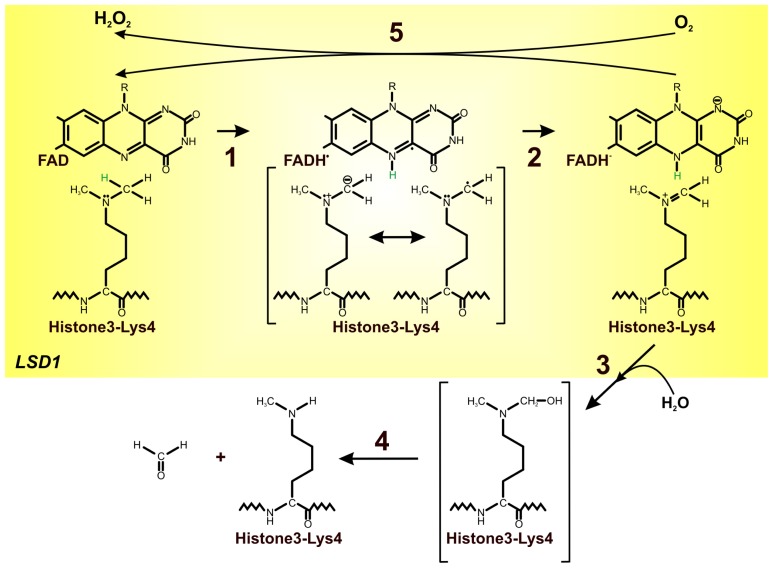
Mechanism of lysine demethylation by LSD1. Simultaneous amine oxidation and flavin reduction are followed by the re-oxidation of flavin by one equivalent of molecular oxygen, producing stoichiometric hydrogen peroxide along with the formaldehyde as the demethylation by-product [48,52]. Flavin dependent amine oxidase (LSD1) catalyzes the flavin-mediated two-electron oxidation of the methylated lysine; it leads to the formation of the corresponding imine molecule with the concomitant reduction of the flavin cofactor (**1**, **2**); following the imine formation, hydration to the N,O-hemiacetal and subsequent collapse to formaldehyde and amine takes place (**3**, **4**); reduced FAD can be re-oxidized by molecular oxygen to hydrogen peroxide (**5**) [47,48,56,62,64], modified.

The catalytic turnover of the lysine-specific histone demethylases (LSD1/2) proceeds as expected for FAD-dependent amine oxidases. Simultaneous amine oxidation and flavin reduction are followed by the re-oxidation of flavin by one equivalent of molecular oxygen, producing stoichiometric hydrogen peroxide along with formaldehyde as the demethylation by-product [48,52]. The first step in the reaction catalyzed by these enzymes is the flavin-mediated two-electron oxidation of the methylated lysine. It leads to the formation of the corresponding imine molecule with the concomitant reduction of the flavin cofactor [47]. Following the imine formation, hydration to the *N*,*O*-hemiacetal and subsequent collapse to formaldehyde and amine takes place [52]. Reduced FAD can be re-oxidized by molecular oxygen with formation and release of hydrogen peroxide which makes the enzyme available for a new catalytic cycle (Figure 4) [49]. On the basis of changes in the flavin optical spectrum it was shown that flavin oxidation is rapid [52]. Baron *et al.*[58] highlighted the role of Lys661 as the “entry residue” which channel the oxygen into the active center of LSD1 and suggested its catalytic function in oxygen activation through the electrostatic stabilization of the superoxide-flavin semiquinone pair, which is thought to transiently form during the electron-transfer process underlying flavin re-oxidation [58].

The major unraveled portion of the oxidative conversion of the amine to the imine is the reductive half-reaction, which involves the irreversible CH bond cleavage. To date, three major mechanisms for this reaction have been discussed: hydride transfer mechanism, polarnucleophilic mechanism, and single electron mechanism [52,62]. Hydride transfer mechanism involves a direct transfer of a hydride from the substrate α-carbon to flavin without the intermediacy of carbanion (Figure 4). In contrast, the polar nucleophilic mechanism and a radical mechanism require additional intermediates, an adduct intermediate and radical intermediate, preceding the CH cleavage [62,63]. The absence of detectable intermediates between oxidized and reduced FAD, as well as the unfavorable energetic for the polar nucleophilic mechanism and radical mechanism, suggests that the rate-limiting reductive half-reaction of LSD1 employed the direct hydride transfer mechanism [62].

A significant number of lysine and arginine residues on the histone tails and various methylation levels which can be generated at each of these sites, provide tremendous regulatory potentials for chromatin modifications [52,55,65,66]. Although LSD1 alone is capable of demethylating H3K4 in peptides, demethylation of H3-K4 in nucleosomes by LSD1 requires corepressor CoREST. CoREST and LSD1 form very tight and stabilizing complex which does not dissociate even at very high ionic strength [50,58]. This complex is an exceptionally relevant target for epigenetic drugs because LSD1 is overexpressed in many solid tumors such as breast, colon, neuroblastoma, bladder, small cell lung, blood, and prostate cancers [67]. Moreover, LSD1 may play an important role in facilitating androgen receptor-mediated transcriptional regulation [48,49,65]. Recent reports suggest that increased LSD1 expression in prostate tumors correlates significantly with relapse during therapy [57,65].

LSD1 might also act on a non-histone substrate such as p53 which activates the expression of genes involved in either cell cycle arrest or apoptosis in response to DNA damage. Demethylation of methylated p53 prevents it from binding DNA and leads to inactivation of p53 target genes [51,57,68]. It has been shown that LSD1 also participates in the repression of E-cadherin, a molecule mediating cell–cell interaction and migration. Downregulation of this factor generally correlates with poor prognosis of tumor and metastasis. Although LSD1 could have an oncogenic effect, it could behave as a tumor suppressor [69]. Studies of Wang *et al.*[54] indicated a role for LSD1 in suppressing breast cancer metastasis by repressing TGFβ1 expression. LSD1 expression was significantly lower in tumor samples compared to adjacent normal tissue and the level of LSD1 expression was negatively correlated with the level of TGFβ1 expression.

Flavin-dependent histone demethylases represent potential targets for chemotherapy [52,55,66,69]. Such inhibitors as tranylcypromine ((±)-*trans*-2-phenylcyclopropyl-1-amine HCl) are of potential relevance for the treatment of promyelocytic leukemia and may be used to block tumor progression [53,55,69]. It was shown that this nonselective MAO inhibitor can be covalently attached to FAD N(5) and can act as a sort of plug which fills the substrate-binding cleft of LSD1. Moreover, Yang *et al.*[66] reveled that phenyl group of the FAD-tranylcypromine adduct in LSD1 is located in a large hydrophobic pocket and does not form extensive contacts with the surrounding residues. It suggests that tranylcypromine analogs with hydrophobic substitutions on the phenyl ring might be more potent inhibitors of LSD1 [70]. Culhane *et al.*[70,71] showed inhibition of LSD1 by propagyl-Lys-derivatived peptide and phenelzine. Schulte *et al.*[72] observed that treatment of neuroblastoma cell lines with the pargyline, clorgyline, or tranylcypromine impaired growth of neuroblastoma cells. Reduced viability of these cells was correlated with increased global dimethylation of lysine 4 in histone 3 [72]. Karytinos *et al.*[50] revealed that tranylcypromine is also an LSD2 inhibitor, while other monoamine oxidase covalent inhibitors such as pargyline, deprenyl, and rasagiline, did not exert any inhibitory effect on LSD2.

## 5. The Role of Riboflavin Supplements in the Prevention of Cancer

Dietary intake of riboflavins could play an important role in the development and progression of cancer. Riboflavin deficiency interferes with the metabolism of other B vitamins, through flavin coenzyme activity. As was already mentioned, riboflavin is the precursor of FAD, the cofactor for methylenetetrahydrofolate reductase, and, by that means, its status influences the metabolism of folates and, consequently, the risk of cancer [2].

Free riboflavin is present in food in small amounts, while most of it occurs as one of its coenzyme derivatives: FAD and FMN. However, flavins which are covalently bound to the enzymes do not appear to be available for absorption, which occurs through carrier-mediated transport process predominantly in the proximal small intestine but also in the colon [1].

The recommended dietary allowance for riboflavin is 1.1 and 1.3 mg/d for women and men, respectively [73]. As cereals, milk, dairy products, meat, and fatty fish, as well as fruits and dark-green leafy vegetables are good sources of riboflavin intake [2,73], in Western countries, riboflavin deficiency is uncommon. However, deficiency is possible for children, adults who avoid drinking milk, and athletes with concerns about body weight [73,74]. Riboflavin deficiency is endemic in many developing countries in populations which exist on diets lacking dairy products and meat. However, riboflavin synthesized by bacterial metabolism in the colon might be an important source of this vitamin [1].

He *et al.*[75] investigated the influence of riboflavin-fortified salt nutrition on the risk of esophageal cancer—the sixth most common cancer on the globe—which causes the highest mortality among the Chinese, whose diet is poor in riboflavin. It has been shown that riboflavin was a protective factor for this type of cancer incidence [75]. Association between dietary intakes of riboflavin and colorectal, cervical, breast, and prostate cancer incidence and mortality was also found [2,4–6,76].

Riboflavin captures reactive metabolites like tamoxifen and carcinogens to form a complex, thereby preventing formations of DNA adduct, preventing DNA methylation, and maintaining genomic stability. It is speculated that it protects against DNA damage by increasing the levels of poly(ADP-ribose) polymerase [6]. Premkumar *et al.*[6] determined the influence of riboflavin supplementation (10 mg one dose per day) on the breast cancer patients undergoing tamoxifen therapy. It is especially important because cancer patients are exposed to high levels of DNA-damaging agents and they may also reveal cancer cachexia connected with chemotherapy. Additionally, cancer cells with inhibited repair-associated enzymes cannot be efficiently repaired, which leads to the cell death [6]. It was shown that riboflavin binds chemioterapheutics and carcinogens to form a complex, and, in this way, prevent formation of DNA adducts and DNA methylation, while maintaining genomic stability. Moreover, riboflavin supplementation influences induction of repair enzymes such as poly(ADP-ribose) polymerase, DNA polymerase beta and DNA ligase [6].

Riboflavin is a cofactor for 5,10-methylenetetrahydrofolate reductase. Low intake of this vitamin reduces the metabolic activity of MTHFR and thus contributes to colorectal carcinogenesis. MTHFR c.*677C* > *T* polymorphism also affects enzyme activity and therefore can be associated with reduced colorectal cancer risk with adequate riboflavin and folate status [4,5,77]. Such association was observed by Powers [4]. In this study, subjects were divided into three groups: placebo group, low-dose folate group (400 μg folic acid and 5 mg riboflavin), and high-dose folate group (1.2 mg folic acid). The riboflavin supplementation enhanced the systemic response to low-dose folic acid in the patients with at least one *T* allele [4]. Powers *et al.*[5] suggested that riboflavin can partially compensate for the reduction in 5,10-methylenetetrahydrofolate reductase activity in heterozygotes and homozygotes for the c.*677C* > *T* mutation, through its cofactor role for this enzyme. McNulty *et al.*[37] confirmed in their study that persons carrying the *TT* genotype for MTHFR are particularly sensitive to riboflavin status to maintain adequate folate status and to prevent the accumulation of homocysteine. De Vogel *et al.*[77] studied the relationship between riboflavin intake and colorectal cancer risk. They showed that riboflavin level was associated with decreased proximal colon cancer risk among women. Gender-specific cancer risk can most likely be connected with higher sensitivity of proximal colon cells in women on riboflavin level, which indirectly leads to differences in DNA hypomethylation between men and women [77].

No association between dietary intake of riboflavin and prostate cancer was detected by Basset *et al.*[2]. There was a suggestive evidence of an inverse U-shaped relationship between incidence and both riboflavin and folate intake and between mortality and riboflavin intake. Additionally, significant interaction between alcohol and riboflavin was observed [2].

## 6. Conclusions

The effect of dietary deficiency of riboflavin on the tumor growth was first noted in 1943. Since then, our knowledge about complex interactions between riboflavins and various enzyme functions, as well as regulation of metabolism, has increased significantly. Now, it is known that the distribution of riboflavin, FMN, and FAD in tissues is under strict metabolic control and the flavin-dependent enzymes play an important role, e.g. in demethylation of histons and stabilization of homocysteine level in one-carbon metabolism. These enzymes are also engaged in biosynthesis of plant secondary metabolites such as glucosinolates involved in detoxification of carcinogenic and/or mutagenic compounds. Therefore from a public health perspective, it is important to identify factors which may be an effective strategy for the prevention of cancer, including riboflavin supplementation.
